# Utilization of surgical safety checklists by urological surgeons in Germany: a nationwide prospective survey

**DOI:** 10.1186/s13037-015-0082-5

**Published:** 2015-11-10

**Authors:** Hendrik Borgmann, Sarah Helbig, Michael A. Reiter, Tanja Hüsch, David Schilling, Igor Tsaur, Axel Haferkamp

**Affiliations:** Department of Urology and Pediatric Urology, University Hospital Frankfurt, Germany, Theodor-Stern-Kai 7, 60590 Frankfurt, Germany

**Keywords:** Patient safety, Communication, Quality management, Risk reduction, Urology

## Abstract

**Objectives:**

We aimed to investigate the contemporary usage rate and habits of the WHO Surgical Safety Checklist (SSC) in German urological departments.

**Methods:**

We designed a 26-item questionnaire that was sent to all urological departments in Germany. The primary aim of this study was to evaluate the usage rate of the SSC. Secondary aims were to compare perioperative characteristics of users vs. non-users of the SSC and to assess circumstances of the SSC application.

**Results:**

A total of 213 of 234 (91 %) urological departments were users of the SSC, and 21 (9 %) were non-users. SSC users had more often a standard protocol, took less time and had fewer people involved for checking perioperative patient data compared to non-users. Financial budgeting for the SSC existed in 55 (24 %) departments and for patient safety in 73 (32 %) departments.

**Conclusions:**

The usage rate of the SSC in urological departments in Germany is high despite restricted financial budgeting. Users of the SSC profit by saving time and manpower for checking perioperative patient data.

**Electronic supplementary material:**

The online version of this article (doi:10.1186/s13037-015-0082-5) contains supplementary material, which is available to authorized users.

## Background

Surgery is one of the most complex health interventions to be delivered. An estimated 234 million people require surgical treatment every year for different medical reasons [1], and adverse events in surgery were reported to occur in 14 % of patients [2]. Among all types of surgery, the rate of preventable adverse events was 71 % for transurethral resection of the prostate or bladder, which was the second most common of all operations and thus seems to possess significant potential for improvement [3]. Urology as a surgical discipline implies various dangers for patient safety because it is a high-volume and a high-technology discipline. Instruments used in endourology or in robotic surgery could endanger the success of an operation due to malfunctions of the technical system [4]. An investigation on wrong-site surgery found that of 126 wrong site surgeries reported, 14 (11 %) were urological [5]. The Joint Commission on Accreditation of Healthcare Organizations identified three key components to reduce the risks of wrong-site surgery: preoperative verification, site marking, and a timeout in the operating room, ensured by a safety checklist [5].

The Safe Surgery Saves Lives Study Group at the World Health Organization published a perioperative surgical safety checklist (SSC) in 2008 [6]. The introduction of the SSC in eight hospitals around the world was associated with a reduction in deaths from 1.5 % to 0.8 % and in major complications from 11.0 % to 7.0 % [7]. In a following study, de Vries et al. reported that implementation of a comprehensive checklist in hospitals with a high standard of care was associated with a reduction in postoperative complication rate from 27.3 % to 16.7 % [8]. Despite the existing evidence for the use of the SSC, little is known about its real usage rate. To date, there are no reports in the medical literature on the usage rate of the SSC for the field of urology. Furthermore, usage habits of the SSC and reasons and circumstances of non-users of the SSC have not been explored.

The objective of this study was to evaluate the usage rate of the SSC in urological departments in Germany. Secondary aims were to compare perioperative characteristics of users vs. non-users of the SSC and to assess circumstances of the SSC application.

## Methods

### Development of the survey

**Fig. 1:**
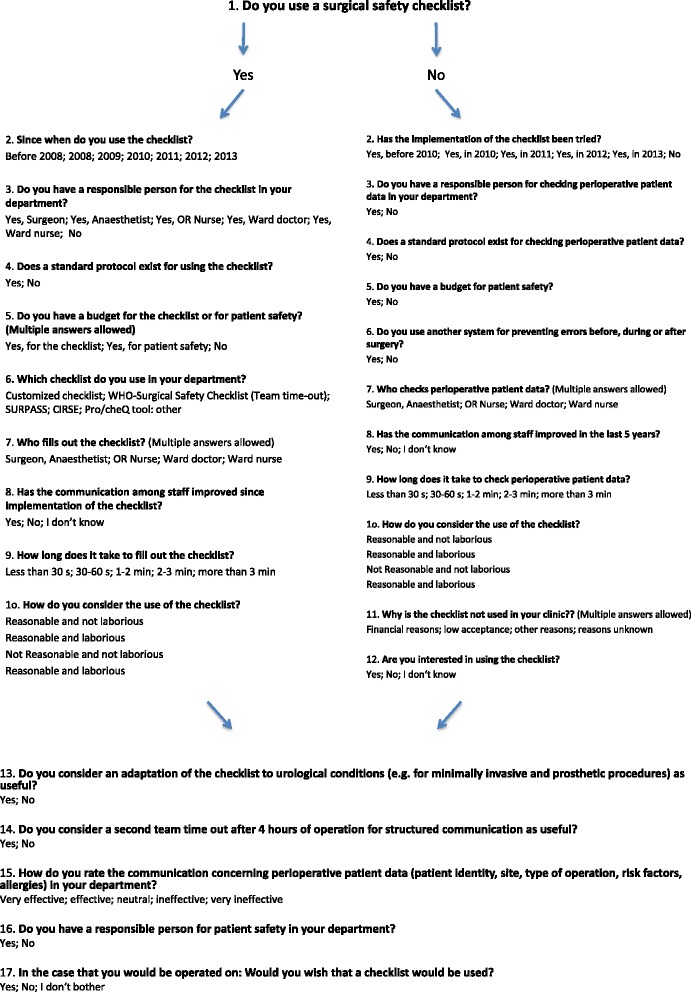
Design of 26-item survey. Design of the 26-item survey that was sent out to 332 urological departments

The survey was designed and conducted according to reporting guidelines for surveys found on equator-network.org, an international initiative providing robust reporting guidelines [9, 10]. The first step for development of the survey was item generation. A literature search on Medline was done using the terms “surgical safety checklist” and “patient safety” in order to identify key topics concerning the use of the SSC. The survey contained 26 items, which were arranged on 4 pages. The response formats were closed and included binary, nominal and ordinal measurements. The complete survey design is depicted in Fig. 1. The survey starts with the central question of whether the responder uses a SSC or not. Depending on the answer, it continues with 9 questions for the user-group and 11 questions for the non-user group. The survey ends for both groups with 5 questions. The survey was then transferred to an independent online tool on www.surveymonkey.com and was approved by an institutional review board. The target populations were the urological departments in Germany. Email and postal addresses of all departments were collected from the database of the German Society of Residents in Urology. Since the number of 332 departments was reachable for this survey, there was no sampling done. The study was conducted in accordance with the Ethical Issues in Patient Safety Research published by the World Health Organization.

### Administration of the survey

Before administration, the survey was tested for usability and technical functionality by doctors from the investigators’ department. It was then sent out to the 332 urological departments via email containing the web link to the online-survey. Once the survey was started an answer to every question was mandatory until completion. The timeframe for data collection via email distribution of the survey was 2 weeks. In order to increase the response rate, the survey was printed out and sent to all 332 urological departments as a paper version. Enclosed was a note not to fill out the paper survey if the online version had already been completed. The timeframe for data collection via postal distribution of the survey was 8 weeks.

### Statistical analysis

Statistical calculations were performed using Statistical Package for the Social Sciences 22.0 software (SPSS Inc., Chicago, IL, USA). The groups of users of the SSC and non-users of the SSC were compared with regard to different parameters. To test whether a standard protocol for checking perioperative patient data was used more frequently by users of the SSC or by non-users of the SSC, the χ^2^ test was applied. The Mann–Whitney U-test was used to assess differences concerning how many people were engaged in and how much time was consumed for checking perioperative patient data. A *p*-value of less than 0.05 was considered significant.

## Results

### Use and practicability of surgical safety checklist

**Fig. 2:**
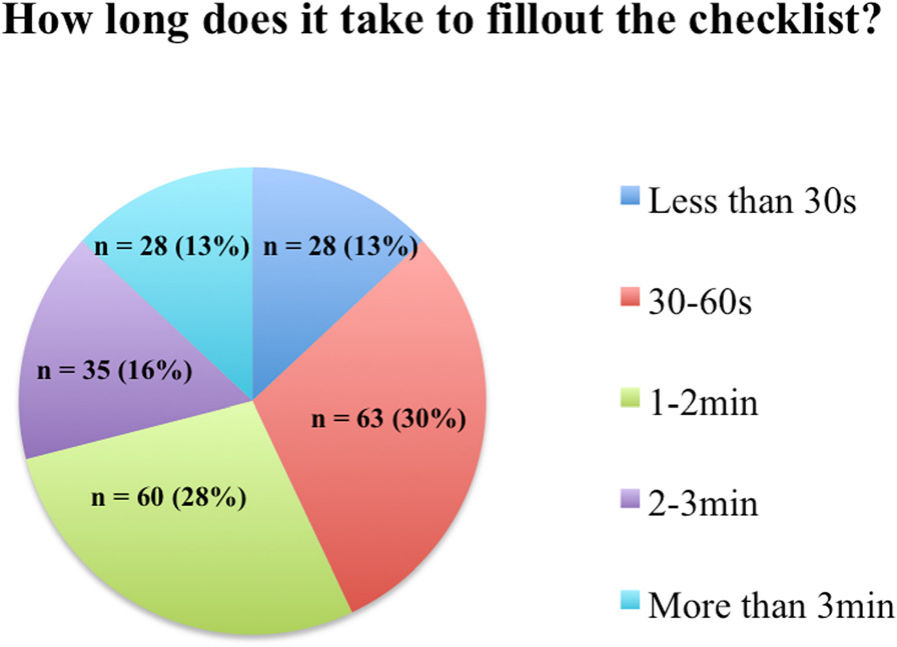
Time consumption for surgical safety checklist application. Estimated time consumption for application of a surgical safety checklist from 213 users of a checklist

Overall, 234 of 332 chairmen responded to the survey resulting in a response rate of 70 %. Of these, 119 (36 %) responded to the online version and 115 (35 %) to the paper version of the survey. Of the 234 responders, 213 (91 %) were users and 21 (9 %) were non-users of the SSC. The users of the SSC found it reasonable and not very laborious (79 %) rather than reasonable and laborious (19 %) or unreasonable and laborious (2 %). The estimated time consumption for completion of the SSC is shown in Fig. 2. Anesthisiologists (87 %), surgeons (85 %), surgical nurses (75 %), ward nurses (37 %) and ward doctors (28 %) were integrated in filling out the SSC.

### Characteristics of users and non-users of the SSC

Differences found between the groups of users and non-users of the SSC are listed in Table 1. Users of the SSC (95 %) more often had a standard protocol for checking perioperative patient data than non-users (76 %), and this difference was statistically significant (*p* = 0.001). In addition, checking perioperative patient data took less time (*p* < 0.001), and fewer people were involved (*p* = 0.008) in the user group than in the non-user group. The reason for non-usage of the SSC was seldom for financial (10 %) or acceptance (24 %) matters and remained unclear (24 %) or of other nature (43 %) in most of the cases.

**Table 1:** Comparison of users and non-users of the surgical safety checklist

Users of the checklist (*n* = 213)			Non-Users of the checklist (*n* = 21)			Statistical Difference
Since when do you use the checklist?			Has the implementation of the checklist been tried?			
Answer Options	Number	Per Cent	Answer Options	Number	Per Cent	
before 2008	36	16.9 %	yes, before 2010	0	0 %	
2008	16	7.5 %	yes, in 2010	1	4.8 %	
2009	22	9.5 %	yes, in 2011	3	14.3 %	
2010	40	17.3 %	yes, in 2012	2	9.5 %	
2011	50	23.5 %	yes, in 2013	4	19.0 %	
2012	39	18.3 %	no	11	52.4 %	
2013	10	4.7 %				
Does a standard protocol exist for using the checklist?			Does a standard protocol exist for checking perioperative patient data?			
Answer Options	Number	Per Cent	Answer Options	Number	Per Cent	
Yes	202	94.8 %	Yes	16	76.2 %	* *P* = 0.001
No	11	5.2 %	No	5	23.8 %	
Who fills out the checklist?			Who checks perioperative patient data?			
Answer Options	Number	Per Cent	Answer Options	Number	Per Cent	
Surgeon	180	84.5 %	Surgeon	20	95.2 %	
Anaesthetist	185	86.9 %	Anaesthetist	20	95.2 %	
OR Nurse	158	74.2 %	OR Nurse	15	71.4 %	
Ward Doctor	60	28.2 %	Ward Doctor	11	52.4 %	
Ward Nurse	78	36.6 %	Ward Nurse	9	42.9 %	
People involved per case	3.1		People involved per case	3.6		* *P* < 0.001
How long does it take to fill out the checklist?			How long does it take to check perioperative patient data?			
Answer Options	Number	Per Cent	Answer Options	Number	Per Cent	
Less than 30s	28	13.1 %	Less than 30s	2	9.5 %	* *P* = 0.008
30-60s	63	29.6 %	30-60s	1	4.8 %	
1-2 min	60	28.2 %	1-2 min	7	33.3 %	
2-3 min	35	16.4 %	2-3 min	4	19.0 %	
More than 3 min	27	12.7 %	More than 3 min	7	33.3 %	
How do you consider the use of the checklist?			Why is the checklist not used in your clinic?			
Answer Options	Number	Per Cent	Answer Options	Number	Per Cent	
Reasonable and not laborious	169	79.3 %	financial reasons	2	9.5 %	
Reasonable and laborious	40	18.8 %	low acceptance	5	23.8 %	
Not reasonable and not laborious	0	0.0 %	other reasons	9	42.9 %	
Not reasonable and laborious	4	1.9 %	reason unknown	5	23.8 %	
			Are you interested in using the checklist?			
			Answer Options	Number	Per Cent	
			Yes	16	76.2 %	
			No	3	14.3 %	
			I don’t know	2	9.5 %	

* indicates a statistically significant difference between both groups

### Resources for SSC and potential for improvement

**Fig. 3:**
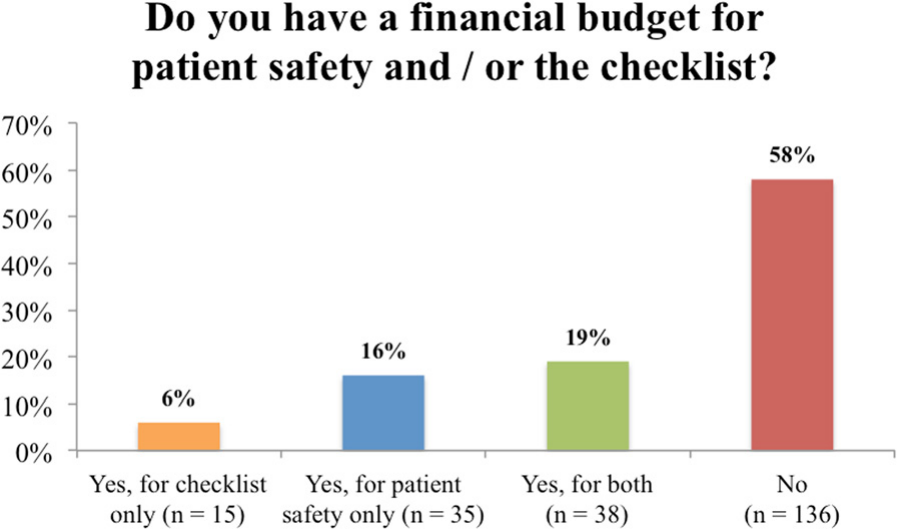
Financial budget. Financial budget of urological departments for a surgical safety checklist and/or patient safety

Figure 3 shows the financial budget of the urological departments for patient safety. Overall, 136 of 234 departments (58 %) had no financial budget for patient safety at all. A budget for the SSC was provided for 15 of 234 departments (6 %). An assigned person in charge of patient safety was present in 162 of 234 (69 %) departments. A second team timeout after 4 h of operation time was reasonable for a minority (63/234, 27 %) of the departments. However, an adaptation of the SSC for specific procedures in minimally invasive and prosthetic surgery was considered to be useful by the majority (148/234, 63 %) of the responders.

## Discussion

A survey on the use of the SSC was sent out to all urological departments in Germany. The results of a representative sample showed a high, but incomplete, usage rate of the SSC of 91 %. The users of the SSC needed less time and fewer people to check perioperative patient data than the non-users. An improvement of the SSC through adaptation to specific procedures in minimally invasive and prosthetic urological surgery was found to be useful by the majority of departments.

The usage rate of the SSC was 91 % in our series of urological departments in Germany. Recent surveys reported a slightly lower usage rate in Switzerland of 79 % [11] and in Ireland of 78 % [12]. The SSC has been mandated for use in surgical procedures in several countries including Sweden, the United Kingdom, and the USA. In England and Wales it has been modified by the National Patient Safety Agency in order to adjust it to national structures and processes. Examples of different versions of the SSC are shown in Additional file 1. Studies on the 30-day morbidity and mortality between countries with overall and fractional use the SSC might shed light on whether a mandated use of the SSC is beneficial.

Although the usage rate of the SSC found for urological departments in Germany was high this does not automatically go along with compliance to the SSC, which represents an unresolved conundrum in the current age of safety checklists. A recent prospective observational study of 854 surgeries performed in Colorado revealed a uniformly poor compliance with respect to assessment of case duration, blood loss, anesthesiologists’ concerns, or display of essential imaging [13]. Moreover, active participation by physicians was observed in only 71 % of surgeries [13]. Our survey respondees claimed higher percentages for involvement of anesthesiologists (87 %) and surgeons (85 %), however the crucial distinction of use of the SSC and compliance to the SSC has to be kept in mind when interpreting our findings.

The time consumption for checking perioperative patient data was lower for the group of users of the SSC than for non-users. Our results of an average time consumption of less than 2 min for completion of the SSC are in agreement with the time consumption found by Taylor et al. [14]. Moreover, an observational study found that preoperative safety briefings did not delay the operating theatre start time [15]. The perception of the users of the SSC was very positive in our population. 98 % found the SSC to be meaningful, and only 21 % found it to be laborious. Accordingly, Norton et al. reported that 89 % of hospital staff believed that the checklist has improved patient safety in the perioperative environment [16].

Only 32 % of departments had a financial budget for patient safety, and only 24 % had a budget for the SSC. In this context, the question of cost effectiveness of the SSC must be discussed. Costs of implementing a checklist mostly involve checklist development and modification, formal staff notification, training, and additional operating room time. In 2010, Semel et al. performed a hypothetical decision analysis of the checklist introduction [17]. Per-use cost of the SSC was only $11 and it generated cost savings once it prevented at least five major complications, since the cost of a major surgical complication was found to be $11,626 on average [18]. Furthermore, hospitals may realize savings through gains in efficiency by introduction of the SSC. A checklist use in operating rooms resulted in improved nurse retention and a decrease in the number of operations that were cancelled or delayed [19]. Additional evidence suggests that operative briefings may actually decrease disruptions to the surgical workflow [20]. In summary, evidence supports cost-effectiveness of the SSC and thus financial budget should be provided for this purpose.

Despite the described success of the SSC, there is still much to be improved. Since every specialty has its own specific needs, the adaptation of the SSC to urological conditions was considered useful by 63 % of the departments. An example for adaptation of the SSC was presented by Khan et al. who developed a comprehensive checklist to be used in operating theatres with advanced robotic technology [21]. The SSC was also adapted by interventional radiologists according to their specific needs [22]. In our survey, the subspecialties of prosthetic and minimally invasive urological surgery were regarded to be especially suitable for adaptation of the SSC. A second team timeout after 4 h for robotic surgery was proposed by Song et al. in order to further improve patient safety during long-lasting operations [23]. The complication rate has been shown to increase linearly over time with a steep increase for long operations [24]. However, only 27 % of responders in our survey considered the introduction of a second team time out after 4 h of operating time to be useful.

There are some limitations to the findings of our study due to survey methodology. Although the response rate of 70 % was very high for a survey, the findings might not be representative for all urological departments because 30 % did not respond. We assume that users of the SSC tended to answer the survey more frequently than non-users, and thus the real usage rate of the SSC might be slightly lower than 91 %. Furthermore, there is the potential for a double answer from one department for both the online and the paper version of the survey, which were sent out successively. However, the paper version included a note not to fill out the paper survey if the online version had already been completed. Finally, our survey was limited to the use of the SSC and did not monitor the compliance to the SSC in terms of quality and performance level of adhering to all required steps in the protocol.

## Conclusion

We found a high usage rate of the SSC in urological departments in Germany of 91 %, although these departments often lack a budget for the SSC and for patient safety. Users of the SSC found it reasonable and saved time and manpower for checking perioperative patient data compared to non-users. The checklist should be understood not merely as a list of items to be checked off, but as an instrument for the improvement of communication, teamwork, and safety culture in the operating room, and it should be used accordingly.

## Additional file

Additional file 1:
**Examples of different surgical safety checklists used to improve patient safety.** A: World Health Organization Surgical Safety Checklist, B: Surgical Safety Checklist used in Canada, C: Colorado hospital association Surgical Safety Checklist used in Colorado/USA, D: Surgical Safety Checklist used by University Hospital Frankfurt in Germany. (PDF 711 kb)

## References

[1] Weiser TG, Regenbogen SE, Thompson KD, Haynes AB, Lipsitz SR, Berry WR (2008). An estimation of the global volume of surgery: a modelling strategy based on available data. Lancet.

[2] Anderson O, Davis R, Hanna GB, Vincent CA (2013). Surgical adverse events: a systematic review. Am J Surg.

[3] Gawande AA, Thomas EJ, Zinner MJ, Brennan TA (1999). The incidence and nature of surgical adverse events in Colorado and Utah in 1992. Surgery.

[4] Chen CC, Ou YC, Yang CK, Chiu KY, Wang SS, Su CK (2012). Malfunction of the da Vinci robotic system in urology. Int J Urol.

[5] VA strengthens critical patient-safety procedure. Two systems replace traditional paper-based informed-consent process with an electronic time-out checklist integrated into electronic records. Health management technology. 2010;31(4):28–9. 20419958

[6] WHO’s patient-safety checklist for surgery. Lancet. 2008;372(9632):1. doi:10.1016/S0140-6736(08)60964-2. 10.1016/S0140-6736(08)60964-2 18603137

[7] Haynes AB, Weiser TG, Berry WR, Lipsitz SR, Breizat AH, Dellinger EP (2009). A surgical safety checklist to reduce morbidity and mortality in a global population. N Engl J Med.

[8] de Vries EN, Prins HA, Crolla RM, den Outer AJ, van Andel G, van Helden SH (2010). Effect of a comprehensive surgical safety system on patient outcomes. N Engl J Med.

[9] Burns KE, Duffett M, Kho ME, Meade MO, Adhikari NK, Sinuff T (2008). A guide for the design and conduct of self-administered surveys of clinicians. CMAJ.

[10] Eysenbach G (2004). Improving the quality of Web surveys: the Checklist for Reporting Results of Internet E-Surveys (CHERRIES). J Med Internet Res.

[11] Mascherek AC, Schwappach DL, Bezzola P (2013). Frequency of use and knowledge of the WHO-surgical checklist in Swiss hospitals: a cross-sectional online survey. Patient Saf Surg.

[12] Nugent E, Hseino H, Ryan K, Traynor O, Neary P, Keane FB (2013). The surgical safety checklist survey: a national perspective on patient safety. Ir J Med Sci.

[13] Biffl WL, Gallagher AW, Pieracci FM, Berumen C (2015). Suboptimal compliance with surgical safety checklists in Colorado: A prospective observational study reveals differences between surgical specialties. Patient Saf Surg.

[14] Taylor B, Slater A, Reznick R (2010). The surgical safety checklist effects are sustained, and team culture is strengthened. Surgeon.

[15] Ali M, Osborne A, Bethune R, Pullyblank A (2011). Preoperative surgical briefings do not delay operating room start times and are popular with surgical team members. J Patient Saf.

[16] Norton EK, Singer SJ, Sparks W, Ozonoff A, Baxter J, Rangel S (2014). Operating Room Clinicians’ Attitudes and Perceptions of a Pediatric Surgical Safety Checklist at 1 Institution. J Patient Saf.

[17] Semel ME, Resch S, Haynes AB, Funk LM, Bader A, Berry WR (2010). Adopting a surgical safety checklist could save money and improve the quality of care in U.S. hospitals. Health Aff.

[18] Dimick JB, Chen SL, Taheri PA, Henderson WG, Khuri SF, Campbell DA (2004). Hospital costs associated with surgical complications: a report from the private-sector National Surgical Quality Improvement Program. J Am Coll Surg.

[19] Nundy S, Mukherjee A, Sexton JB, Pronovost PJ, Knight A, Rowen LC (2008). Impact of preoperative briefings on operating room delays: a preliminary report. Arch Surg.

[20] Henrickson SE, Wadhera RK, Elbardissi AW, Wiegmann DA, Sundt TM (2009). Development and pilot evaluation of a preoperative briefing protocol for cardiovascular surgery. J Am Coll Surg.

[21] Ahmed K, Khan N, Khan MS, Dasgupta P (2013). Development and content validation of a surgical safety checklist for operating theatres that use robotic technology. BJU Int.

[22] Athreya S, Mikhail M, Reis Welsh S, Muzzafar A, Martin D (2013). Patient safety in interventional radiological procedures: safety checklists and protocols. J Patient Saf.

[23] Song JB, Vemana G, Mobley JM, Bhayani SB (2013). The second “time-out”: a surgical safety checklist for lengthy robotic surgeries. Patient Saf Surg.

[24] Procter LD, Davenport DL, Bernard AC, Zwischenberger JB (2010). General surgical operative duration is associated with increased risk-adjusted infectious complication rates and length of hospital stay. J Am Coll Surg.

