# Supplementary material for: Utilization of surgical safety checklists by urological surgeons in Germany: a nationwide prospective survey
Source: Patient Saf Surg. 2015 Nov 10;9:37. doi: 10.1186/s13037-015-0082-5 (PMC4641404; doi:10.1186/s13037-015-0082-5)
Supplement: Additional file 1: — Examples of different surgical safety checklists used to improve patient safety. A: World Health Organization Surgical Safety Checklist, B: Surgical Safety Checklist used in Canada, C: Colorado hospital association Surgical Safety Checklist used in Colorado/USA, D: Surgical Safety Checklist used by University Hospital Frankfurt in Germany. (PDF 711 kb) [file 13037_2015_82_MOESM1_ESM.pdf]

Surgical Safety Checklist

World Health Organization | Patient Safety
A World Alliance for Safer Health Care

Before induction of anaesthesia

(with at least nurse and anaesthetist)

Has the patient confirmed his/her identity, site, procedure, and consent?

- ☐ Yes

Is the site marked?

- ☐ Yes
- ☐ Not applicable

Is the anaesthesia machine and medication check complete?

- ☐ Yes

Is the pulse oximeter on the patient and functioning?

- ☐ Yes

Does the patient have a:

Known allergy?

- ☐ No
- ☐ Yes

Difficult airway or aspiration risk?

- ☐ No
- ☐ Yes, and equipment/assistance available

Risk of >500ml blood loss (7ml/kg in children)?

- ☐ No
- ☐ Yes, and two IVs/central access and fluids planned

Before skin incision

(with nurse, anaesthetist and surgeon)

- ☐ **Confirm all team members have introduced themselves by name and role.**
- ☐ **Confirm the patient's name, procedure, and where the incision will be made.**

Has antibiotic prophylaxis been given within the last 60 minutes?

- ☐ Yes
- ☐ Not applicable

Anticipated Critical Events

To Surgeon:

- ☐ What are the critical or non-routine steps?
- ☐ How long will the case take?
- ☐ What is the anticipated blood loss?

To Anaesthetist:

- ☐ Are there any patient-specific concerns?

To Nursing Team:

- ☐ Has sterility (including indicator results) been confirmed?
- ☐ Are there equipment issues or any concerns?

Is essential imaging displayed?

- ☐ Yes
- ☐ Not applicable

Before patient leaves operating room

(with nurse, anaesthetist and surgeon)

Nurse Verbally Confirms:

- ☐ The name of the procedure
- ☐ Completion of instrument, sponge and needle counts
- ☐ Specimen labelling (read specimen labels aloud, including patient name)
- ☐ Whether there are any equipment problems to be addressed

To Surgeon, Anaesthetist and Nurse:

- ☐ What are the key concerns for recovery and management of this patient?

This checklist is not intended to be comprehensive. Additions and modifications to fit local practice are encouraged.

Revised 1 / 2009

© WHO, 2009

A: **World Health Organization Surgical Safety Checklist**

FIGURE 1.

Surgical Safety Checklist – Canadian Version for reference only

cpsi Canadian Patient Safety Institute

SURGICAL SAFETY CHECKLIST

www.safesurgerysaveslives.ca

Your Organizational Logo

BRIEFING – Before induction of anesthesia

Hand-off from ER, Nursing Unit or ICU

- ☐ Anesthesia equipment safety check completed
- ☐ Patient information confirmed
  - Identity (2 identifiers)
  - Consent(s)
  - Site and procedure
  - Site, side and level marked
  - Clinical documentation
  - History, physical, labs, biopsy and x-rays
- ☐ Review final test results
- ☐ Confirm essential imaging displayed
- ☐ ASA Class
- ☐ Allergies
- ☐ Medications
  - Antibiotic prophylaxis: double dose?
  - Glycemic control
  - Beta blockers
  - Anticoagulant therapy (e.g., Warfarin)?
- ☐ VTE Prophylaxis
  - Anticoagulant
  - Mechanical
- ☐ Difficult Airway / Aspiration Risk
  - Confirm equipment and assistance available
- ☐ Monitoring
  - Pulse oximetry, ECG, BP, arterial line, CVP, temperature and urine catheter
- ☐ Blood loss
  - Anticipated to be more than 500 ml (adult) or more than 7 ml/kg (child)
  - Blood products required and available
  - Patient grouped, screened and cross matched

BRIEFING *(continued)*

- ☐ Surgeon(s) review(s)
  - Specific patient concerns, critical steps, and special instruments or implants
- ☐ Anesthesiologist(s) review(s)
  - Specific patient concerns and critical resuscitation plans
- ☐ Nurses(s) review(s)
  - Specific patient concerns, sterility indicator results and equipment / implant issues
- ☐ Patient positioning and support / Warming devices
- ☐ Special precautions
- ☐ Expected procedure time / Postoperative destination

TIME OUT – Before skin incision

- ☐ All team members introduce themselves by name and role
- ☐ Surgeon, Anesthesiologist, and Nurse verbally confirm
  - Patient
  - Site, side and level
  - Procedure
  - Antibiotic prophylaxis: repeat dose?
  - Final optimal positioning of patient
- ☐ "Does anyone have any other questions or concerns before proceeding?"

DEBRIEFING – Before patient leaves OR

- ☐ Surgeon reviews with entire team
  - Procedure
  - Important intra-operative events
  - Fluid balance / management
- ☐ Anesthesiologist reviews with entire team
  - Important intra-operative events
  - Recovery plans (including postoperative ventilation, pain management, glucose and temperature)
- ☐ Nurse(s) review(s) with entire team
  - Instrument / sponge / needle counts
  - Specimen labeling and management
  - Important intraoperative events (including equipment malfunction)
- ☐ Changes to post-operative destination?
- ☐ What are the KEY concerns for this patient's recovery and management?
- ☐ Could anything have been done to make this case safer or more efficient?

Hand-off to PACU / RR, Nursing Unit or ICU

PATIENT INFORMATION

Adapted from the WHO Surgical Safety Checklist, © World Health Organization, 2008

Surgical Safety Checklist: Canada
Version 1, January 9, 2009

B: **Surgical Safety Checklist used in Canada**

COLORADO SURGICAL SITE CHECKLIST

Prior to Anesthesia: SIGN IN

Patient has confirmed:
- ☐ Identity (name and DOB)
- ☐ Site
- ☐ Procedure

☐ Consent form is signed by patient

☐ Site marked ☐ NA

☐ H&P is complete and updated

☐ Patient allergies have been reviewed

☐ Diagnostic and radiology test results received

☐ Verify essential imaging is displayed and labeled correctly ☐ NA

☐ Verify antibiotic prophylaxis within 60 minutes of incision (120 minutes for vancomycin/ flouroquinolones) ☐ NA

Does patient require a beta blocker
☐ Yes and administered ☐ No

Does patient require VTE prophylaxis
☐ Yes (boots/anticoagulants ready) ☐ NA

☐ Identify and obtain special necessary medications, equipment, blood or other products ☐ NA

☐ Risk of hypothermia assessed

☐ Verify pre-anesthesia assessment is complete

☐ Verify pulse ox is on patient and functioning

Prior To Incision: TIME OUT

☐ All team members cease activity for TIME OUT

☐ Team members introductions if necessary

Procedure team reviews and affirms:
- ☐ Correct Patient
- ☐ Correct site is marked ☐ NA
- ☐ Correct procedure
- ☐ Correct patient position

☐ Surgeon reviews anticipated events:
- Critical or potential unexpected steps
- Duration of procedure
- Blood loss/blood product availability
- Need for special implants/ equipment

☐ Anesthetist reviews any patient safety concerns

☐ Nursing Team confirms sterility/indicator results

☐ Identify special safety precautions based on patient history or medication use

Prior to Leaving OR: SIGN OUT

Nurse verbally confirms:
- ☐ Name of procedure was recorded
- ☐ The instrument, sponge and needle counts are correct (as applicable)
- ☐ Specimen is labeled (name and DOB)
- ☐ Address any equipment problems that need to be corrected

☐ Proceduralist, anesthetist and nurse review key concerns for patient recovery and management

☐ Discontinue prophylactic antibiotics (if applicable)

☐ VTE prophylaxis administered ☐ NA

☐ Surgical team debriefing if necessary

This checklist is based on the WHO Surgical Safety Checklist developed by the World Health Organization. This checklist is also inclusive of additional Joint Commission requirements and the Surgical Care Improvement Project measures

c|h|a
Colorado Hospital Association

RN Signature: ____________________

Date: __________ Time: __________

C: **Colorado hospital association Surgical Safety Checklist used in Colorado / USA**

UNIVERSITÄTS KLINIKUM FRANKFURT GOETHE-UNIVERSITÄT	Formular<br>**Perioperative Checkliste - TeamTimeOut**	**Qualitäts-management**<br>**Dokumentangaben:**<br>Freigabe: 03.06.2014<br>Version: 018/06.2014<br>ID-Nr.: 5767
Gültigkeitsbereich: Universitätsklinikum Frankfurt		
roXtra-Bearbeiter: Strametz, Reinhard	Autor(en): WHO-Checkliste erweitert mit Mitarbeitervorschlägen	Seite 1 / 1

Patientenetikett	geplante Operation: ______________________________<br>Seite: rechts / links / beidseits / nicht zutreffend / Details: __________<br>Datum der Operation: __________ infektiös: nein / ja: __________<br>besondere präop. Vorbereitung: ______________________________

	Vorbereitung durch Pflege	Pflege Station
	Patientenarmband kontrolliert durch aktives Erfragen der Identität	
	Bettencheck durchgeführt	
	Pflegevorbereitung erfolgt (z.B. Prothesen entfernt, Hautzustand erhoben), Ausnahme(n): ____________	

	Vorbereitung durch Ärztlichen Dienst					Ärztlicher Dienst
	Patient aufgeklärt und Einwilligungen von Arzt und Patient unterschrieben					
	präoperative Diagnostik vollständig und sofern nicht in LA Freigabe Anästhesie erfolgt					
	Blutgruppenbestimmung nicht erforderlich		Blutgruppenbestimmung erfolgt		Anzahl bestellter EKs:	

Handzeichen Pflege ____________________ Handzeichen vorbereitende(r) Arzt/Ärztin ____________________

	Vor Einleitung Anästhesie (wenn nur Lokalanästhesie nur die ersten vier Punkte kontrollieren)			Anästhesisten
	Identitätscheck über Patientenarmband und Patientenauskunft bzw. andere zweite Quelle			
	Bestätigung Prozedur, Körperseite, Einverständnis, Kontrolle Seitenmarkierung			
	Gerätecheck durchgeführt, Monitoring (inbs. SpO_2) funktioniert, Patientenfragen erfolgt (z.B. Nüchternheit, Zähne fest etc.)			
	sofern vorhanden Implantate (z.B. Herzschrittmacher) und Fremdmaterial (Zahnprothese) adäquat berücksichtigt			
	kein/geringer Blutverlust erwartet		Blutverlust >500ml / >7ml/kg KG erwartet → PBM-Checkliste!	Patient Blood Management Wir sind dabei!
	keine schwierige ITN erwartet / keine RSI geplant		schwierige ITN / RSI ? → Ausrüstung & Expertise vorhanden!	

Handzeichen Anästhesist/-in ____________________

	Vor Hautinzision (gemeinsame Auszeit ALLER Beteiligten, OP-Springer liest vor)			OP-Pflege
	alle Teammitglieder vorgestellt (inkl. Praktikanten/Studierenden) oder ALLE untereinander bekannt			
	Anästhesist, Chirurg und OP-Pflege bestätigen: Patientenidentität, Prozedur und Körperseite/Details			
	Prüfung kritischer Punkte: kritische/unerwartete OP-Schritte, Zeit, Blutverlust, ggf. bisherige Probleme während Anästhesie			
	OP-Pflege: Ausrüstung komplett am Tisch vorhanden und einsatzbereit			
	Antibiotikaprophylaxe gegeben:____________________ vor ____ min		nicht erforderlich	
	relevantes Bildmaterial vorhanden und geprüft: richtiger Patient, richtige(s) Bild/Seite		nicht erforderlich	

Handzeichen OP-Springer/-in ____________________

	Vor Verlassen des OPs nach Hautnaht (gemeinsame Auszeit ALLER Beteiligten, letzter Operateur im Saal liest vor)	Operateure
	Bestätigung: richtige Dokumentation der Prozedur und sofern vorhanden richtige Beschriftung aller Präparate	
	Vollständigkeit der Zählkontrolle von Instrumenten, Tüchern/Tupfern und Nadeln	
	postoperatives Management dokumentiert und mit Anästhesie/nachsorgender Einrichtung abgestimmt	

Handzeichen letzte/r Operateur/-in im Saal ____________________

D: **Surgical Safety Checklist used by University Hospital Frankfurt in Germany**
